# CircHIPK3 Promotes Metastasis of Gastric Cancer *via* miR-653-5p/miR-338-3p-NRP1 Axis Under a Long-Term Hypoxic Microenvironment

**DOI:** 10.3389/fonc.2020.01612

**Published:** 2020-08-13

**Authors:** Yue Jin, Xiaofang Che, Xiujuan Qu, Xin Li, Wenqing Lu, Jie Wu, Yizhe Wang, Kezuo Hou, Ce Li, Xiaojie Zhang, Jianping Zhou, Yunpeng Liu

**Affiliations:** ^1^Department of Medical Oncology, The First Hospital of China Medical University, Shenyang, China; ^2^Key Laboratory of Anticancer Drugs and Biotherapy of Liaoning Province, The First Hospital of China Medical University, Shenyang, China; ^3^Liaoning Province Clinical Research Center for Cancer, Shenyang, China; ^4^Key Laboratory of Precision Diagnosis and Treatment of Gastrointestinal Tumors, Ministry of Education, Shenyang, China; ^5^Department of Gastrointestinal Surgery, The First Hospital of China Medical University, Shenyang, China

**Keywords:** circHIPK3, long-term hypoxic microenvironment, HIF-2α, gastric cancer, metastasis

## Abstract

As a vital feature of the microenvironment, hypoxia, especially long-term hypoxia, is known to promote metastasis and lead to poor prognosis in solid tumors. Circular RNAs (circRNAs) participate in important processes of cell proliferation and metastasis in cancers. However, the contribution of circRNAs to metastasis under long-term hypoxia is obscure. In this study, we aim to explore specific functions of circHIPK3 in long-term hypoxia-promoting metastasis of gastric cancer (GC). The hypoxic resistant gastric cancer (HRGC) cell lines we established previously, which were tolerant to 2% O_2_ conditions, were used as the long-term hypoxia model. We found that circHIPK3 was upregulated by HIF-2α in HRGC cells, and circHIPK3 facilitated the migration and invasion ability of HRGC cells. Further investigation proved that circHIPK3 promoted metastasis of HRGC cells directly by interacting with miR-653-5p and miR-338-3p to relieve the suppression of neuropilin 1 (NRP1), resulting in the activation of downstream ERK and AKT pathways. Our study identified oncogene functions of circHIPK3 under a long-term hypoxic microenvironment and the possibility of using circHIPK3 as a potential biomarker of long-term hypoxia in GC. In conclusion, circHIPK3 could promote GC metastasis via the miR-653-5p/miR-338-3p-NRP1 axis under a long-term hypoxic microenvironment.

## Introduction

Gastric cancer is a kind of global malignant tumor, especially in developing countries including China. In China, GC ranks as the fifth most common cancer and the third-ranked leading cause of cancer-related death (1). Even though tremendous advances have been made in diagnosis and treatment strategies in recent years, the prognosis of GC patients remains poor on account of its high relapse and metastatic rates (2). Therefore, exploring novel molecular mechanisms underlying metastasis would provide potential target candidates for prognosis improvement in GC.

Hypoxia, an important microenvironment feature in solid tumors, can promote distant metastasis (3, 4). In a hypoxic microenvironment, hypoxia-inducible factors (HIFs) are upregulated due to the stabilization of HIF-α subunits and play a vital role in tumor progression including angiogenesis, metabolic reprogramming, invasion, and resistance to radiation therapy or chemotherapy (5). Hundreds of genes including VEGFA, Glut1, KLF8, ITGβ1 and etc., transcribed by HIFs are reported to promote metastasis and result in poor prognosis of GC (6–9). However, most of these studies are based on acute hypoxia treatment, while the actual condition inside solid tumors is chronic or cycling hypoxia, which deserves greater concern (10, 11). However, to date, few studies have been focused on long-term hypoxia-promoting tumor metastasis. The limited studies related to long-term hypoxia of tumors reported that slug promoted metastasis of prostate cancer under chronic hypoxia (12); miR-191 induced by chronic hypoxia promoted cell migration in NSCLC (13). Due to the discovery more novel important functions of non-coding RNAs (ncRNAs) including miRNAs, LncRNAs, and circRNAs, participating in tumor progression, we pay special attention in the present work to the role of hypoxia microenvironment-related ncRNAs in GC. In our previous study, we established HRGC cell lines to stimulate the real situation of a long-term hypoxic microenvironment, and found that LncRNA UCA1 was upregulated and promoted the migration of GC cells through the miR-7-5p/EGFR axis under a long-term hypoxic microenvironment (14). However, the biological functions of another subtype of ncRNAs—circRNAs involved in long-term hypoxia-promoting metastatic process of GC remain largely unknown.

Circular RNA (circRNA) is a class of single-strand endogenous ncRNAs formed by 3′ and 5′ joining to form a covalently closed continuous loop (15, 16). Accumulating evidence has shown that circRNAs are essential in the development of various diseases, especially cancers (17). Many circRNAs are reported to play a vital role in tumor metastasis. For example, circNSD2 promoted metastasis of colorectal cancer by targeting miR-199b-5p-mediated DDR1 and JAG1 signaling (18); circPRMT5 promoted metastasis of urothelial carcinoma through sponging with miR-30c (19). However, none of them are related to long-term hypoxia-promoting metastasis. CircHIPK3, an identified circular RNA of 1099 bp in length, is reported to have significant promotional effects on the progression of various cancers including lung cancer, colorectal cancer, and glioma (20–22). However, its function in GC remains ambiguous. It was reported that circHIPK3 could promote proliferation and migration in GC indicating its oncogenic role, while circHIPK3 was downregulated in GC tissues compared to para-carcinoma tissues indicating its tumor-suppressing role (23, 24). The different roles might be due to the strong heterogeneity of GC resulting in the inconsistent effect of circHIPK3 in different specimens. Therefore, the role of circHIPK3 in GC remains to be further studied in detail. Considering that hypoxia might be a crucial reason leading to GC heterogeneity, we aimed to explore the functions and molecular mechanisms of circHIPK3 on long-term hypoxia-promoting metastasis of GC.

In this study, we demonstrated that circHIPK3 was increased under long-term hypoxic microenvironment and could promote metastasis through the miR-653-5p/miR-338-3p-NRP1 axis in GC. These findings elucidated a new mechanism of hypoxia-induced metastasis in GC and revealed the possibility of using circHIPK3 as a new biomarker for long-term hypoxia.

## Materials and Methods

### Patient Tissue Samples

Thirty-one GC patients without therapy before surgery between 2018 to 2019 were enrolled in our study. All the GC and adjacent normal tissues were obtained from operation excision specimens of GC patients in the First Hospital of China Medical University (Shenyang, China). Tissues were promptly frozen in liquid nitrogen and then stored at −80°C. The research was approved by the Ethics Committee of the First Hospital of China Medical University (No. 2019-24-2), and all procedures were conducted according to ethical principles.

### Cell Culture

Human gastric cancer cell lines MGC803 (TCHu84) and BGC823 (TCHu11) were purchased from the Chinese Academy of Sciences (Shanghai, China). These cells were cultured with RPMI-1640 medium containing 10% heat-inactivated fetal bovine serum (FBS) and 1% penicillin-streptomycin. The two long-term HRGC cell lines, MGC803/Hypo and BGC823/Hypo, established from MGC803 and BGC823 in our laboratory (14), were cultured with DMEM containing 10% FBS and 1% penicillin-streptomycin under 2% O_2_ concentration. All the cells were cultured in a 5% CO_2_ humidified incubator at 37°C.

### Reagents and Antibodies

AKT (#9272), phosphorylated (p)-AKT (#9271), p-ERK (#4370), and NRP1 (#3725) antibodies were obtained from Cell Signaling Technology (Danvers, United States). β-actin (sc-47778) and ERK (sc-514302) antibodies were obtained from Santa Cruz Biotechnology (Santa Cruz, United States).

### RNA Isolation and Quantitative Real-Time PCR

Total RNA was isolated with Trizol reagent (Invitrogen, United States) and quantified by measuring the absorbance at 260 nm by nanodrop 2000 (Thermo Fisher Scientific, United States). The reverse transcription reagents were all purchased from TaKaRa (Shiga, Japan). The PrimeScript^TM^ RT reagent Kit (Takara, Japan) was used for mRNA reverse transcription and the One Step PrimeScript^®^ miRNA cDNA Synthesis Kit (Takara, Japan) was used for miRNA reverse transcription. Quantitative real-time PCR was carried out with SYBR Premix Ex Taq II (TaKaRa) and detected using Applied Biosystems^®^ 7500 Real-Time PCR Systems (Thermo Fisher Scientific, United States). 1000 ng RNA was used for cDNA Synthesis and 40 ng cDNA was used for qRT-PCR. The internal control for mRNA and circRNA was 18S and the internal control for miRNA was U6. The *n*-fold change of the RNA expression was calculated using the 2^–ΔΔCt^ method. All primer sequences are listed in Supplementary Table S1.

### Transfection

The specific siRNAs targeted to circHIPK3 and NRP1, miR-653-5p and miR-338-3p mimics or inhibitors, and their corresponding NC, were compounded by JTS Scientific (Wuhan, China). CircHIPK3 overexpression plasmid (pCD25-circHIPK3-GFP) was designed and constructed by Geneseed Biotech Co. (Guangzhou, China). HRGC cells or their parent GC cells (1.0 × 10^5^) were transfected with 0.1 μM siRNAs, 0.1 μM miRNA mimics/0.15 μM inhibitors, or 1 mg/L plasmids using jetPRIME^®^ Transfection Reagent according to manufacturer’s instructions. The sequences of all siRNAs or mimics/inhibitors are shown in Supplementary Table S1.

### Transwell Migration and Invasion Assay

Transwell chambers (Corning, NY, United States) were plated into a 24-well plate. For migration assay, 2 × 10^4^ cells were plated within 200 μL serum-free medium onto the upper chamber and 500 μL medium with 10% FBS was added to the lower chamber. After incubating for 24 h, the chambers were fixed with methanol and then stained with Wright-Giemsa dye. The stained cells were counted and analyzed statistically. For invasion assay, except for pre-coating the chamber with 50 μL diluted-matrigel before the cells were plated onto the upper chamber, other steps were as outlined for the aforementioned migration assay.

### Western Blot Assay

All treated cells were lyzed by 1% Triton lysis buffer. After quantification, the protein samples were mixed with 3 × loading buffer. The prepared samples were separated by SDS-polyacrylamide gel electrophoresis and then transferred onto PVDF membranes (Millipore, United States). Next, the PVDF membranes were blocked with 5% skimmed milk in TBST buffer, and then incubated with the primary antibodies overnight at 4°C. The following day, the membranes were incubated with the secondary antibodies. Finally, the membranes were examined with enhanced chemiluminescence reagent and visualized using the Electrophoresis Gel Imaging Analysis System (DNR Bio-Imaging Systems, Israel).

### RNA Immunoprecipitation

RNA immunoprecipitation (RIP) assays were executed by the Magna RIP RNA-Binding Protein Immunoprecipitation Kit (Millipore, Burlington, MA, United States) according to manufacturer’s protocols. HRGC cells were lysed in lysis buffer and then incubated with RIP immunoprecipitation buffer which contained magnetic beads pre-incubated with the anti-AGO2 and anti-IgG (Millipore, United States). RNA was purified from RNA-protein complex and detected by qRT-PCR.

### Luciferase Reporter Assay

Hypoxic resistant gastric cancer cells (to a total number of 2.5 × 10^4^) were co-transfected with pmirGLO-circHIPK3-WT and pmirGLO-circHIPK3-MUT (RiboBio, Guangzhou, China) or pmirGLO-NRP1-WT and pmirGLO-NRP1-MUT (OBIO, Shanghai, China) and miR-NC or miR-653-5p or miR-338-3p mimics (JTS Scientific, Wuhan, China). Twenty-four hours later, the luciferase activity of cell lysates was examined by a Dual Luciferase Reporter System (Promega, United States).

### RNA Pull Down Assay

Biotinylated-circHIPK3 and control probes were synthesized by RiboBio (Guangzhou, China). A total of 1.0 × 10^7^ HRGC cells were washed by cold PBS, and then lysed and sonicated. The biotinylated-circHIPK3 and control probes were used for incubation with C-1 magnetic beads (Life Technologies) at 25°C for 2 h. The cell lysate was incubated with the biotinylated-circHIPK3 or control probe at 4°C overnight. Then the beads were washed by buffer and miRNAs were extracted using Trizol reagent and analyzed by qRT-PCR assay. The sequence of circHIPK3 probe was biotin-5′-ACTTGTGAGGCCATACCTGT AGTACCGAGATT-3′; the sequence of control probe was biotin-5′-CGACTTTGGCTTGTTCTGGCCTGCATGACTGTTGAAA TGT- 3′.

### Statistical Analysis

The data are all shown as mean ± SD with three independent experiments. An unpaired Student’s *t*-test was used to analyze the statistical differences between two groups and *p-*value < 0.05 was regarded as indicative of significance.

## Results

### CircHIPK3 Was Upregulated by HIF-2α in HRGC Cells

**FIGURE 1:**
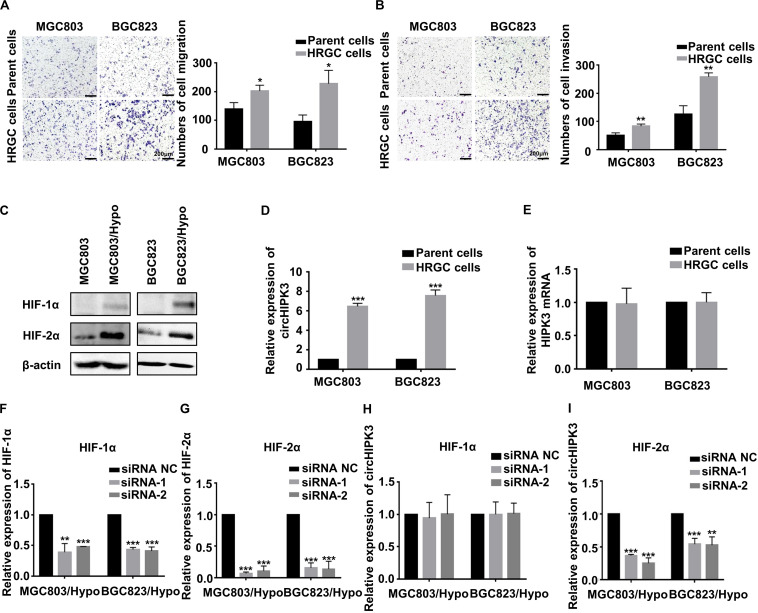
CircHIPK3 was upregulated by HIF-2α in HRGC cells. **(A,B)** The migration and invasion ability of HRGC cells and their parent GC cells was examined by transwell assay (original magnification, 100×). The columns on the right are quantified by counting three fields, and presented as the mean ± standard deviation. *p < 0.05, **p < 0.01. **(C)** The protein expression of HIF-1α and HIF-2α in HRGC cells compared with their parent GC cells was detected by western blot. β-actin was used as an internal control. **(D,E)** The relative expression of circHIPK3 and linear HIPK3 mRNA in HRGC cells and their parent GC cells was detected by qRT-PCR. **(F,G)** The knockdown efficiency of HIF-1α or HIF-2α in HRGC cells was detected by qRT-PCR. **(H,I)** The relative expression of circHIPK3 in HRGC cells after transfected with HIF-1α or HIF-2α siRNAs was detected by qRT-PCR. Data are presented as the mean ± SD of three independent experiments. *p < 0.05, **p < 0.01, ***p < 0.001. 18S was used as an internal control for all qRT-PCR experiments.

Firstly, the migration and invasion capability, and HIF-1α and HIF-2α protein, two important hypoxia-related markers in HRGC cells were compared with those in their parent GC cells. As a result, the migration and invasion ability of HRGC cells was notably enhanced, and HIF-2α was remarkably upregulated whereas HIF-1α was merely slightly upregulated in HRGC cells, which was similar to the findings of our previous research (14) (Figures 1A–C). Then, circHIPK3 expression levels in HRGC cells and their parent GC cells were examined by qRT-PCR analysis, and the result showed that circHIPK3 expression in HRGC cells was notably upregulated more than 5-fold over that in their parent GC cells, while the expression of linear HIPK3 mRNA was practically unchanged under the long-term hypoxic microenvironment (Figures 1D,E). To explore whether HIF-1α or HIF-2α is involved in hypoxia-induced circHIPK3 upregulation, HIF-1α and HIF-2α were knocked down. The result of qRT-PCR showed that HIF-2α knockdown (KD) but not HIF-1α KD decreased the expression of circHIPK3 in HRGC cells, indicating that HIF-2α mainly contributed to circHIPK3 upregulation in GC under a long-term hypoxic microenvironment (Figures 1F–I).

### CircHIPK3 Promoted Migration and Invasion of HRGC Cells

**FIGURE 2:**
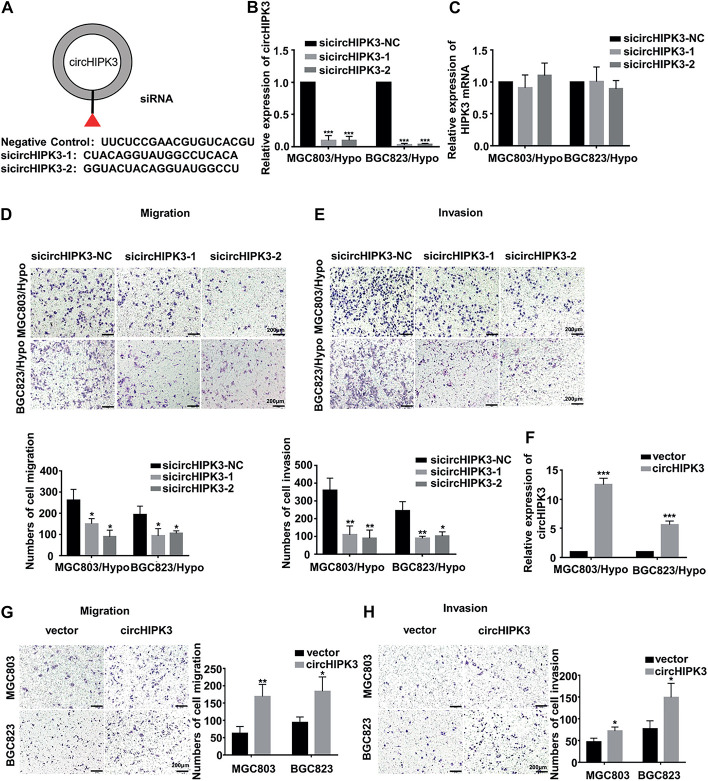
CircHIPK3 promoted migration and invasion of HRGC cells. **(A)** The sequence of two siRNAs targeted to back-splicing site of circHIPK3 and the negative control siRNA. **(B,C)** The relative expression of circHIPK3 and linear HIPK3 mRNA in HRGC cells after transfected with negative control siRNA (siNC) or circHIPK3 siRNAs was detected by qRT-PCR. 18S was used as an internal control. **(D,E)** The migration and invasion ability of HRGC cells after transfected with siNC or circHIPK3 siRNAs was examined by transwell assay (original magnification, 100×). The columns on the down panels are quantified by counting 3 fields, and presented as the mean ± standard deviation. *p < 0.05, **p < 0.01, ***p < 0.001. **(F)** The overexpression efficiency of circHIPK3 in MGC803 and BGC823 cells was detected by qRT-PCR. 18S was used as an internal control. **(G,H)** The migration and invasion ability of MGC803 and BGC823 cells after transfected with circHIPK3 overexpression plasmids and empty vectors was examined by transwell assay (original magnification, 100×). The columns on the right are quantified by counting three fields, and presented as the mean ± standard deviation. *p < 0.05, **p < 0.01, ***p < 0.001. Data are presented as the mean ± SD of three independent experiments. *p < 0.05, **p < 0.01, ***p < 0.001.

To identify whether circHIPK3 is involved in long-term hypoxia-promoting metastasis of GC cells, circHIPK3 was transiently knocked down with nearly no expression change in parent gene HIPK3 (Figures 2A–C), and transwell assays were then performed. It was shown that circHIPK3-KD significantly restrained the migration and invasion capability of both MGC803/Hypo and BGC823/Hypo cells (Figures 2D,E). On the contrary, when overexpressing circHIPK3 in MGC803 and BGC823 cells to imitate a long-term hypoxic microenvironment (Figure 2F), the migration and invasion ability was significantly increased (Figures 2G,H). All of these results indicated that circHIPK3 promoted GC metastasis under a long-term hypoxic microenvironment.

### CircHIPK3 Promoted Migration and Invasion of HRGC Cells by Sponging With miR-653-5p and miR-338-3p

**FIGURE 3:**
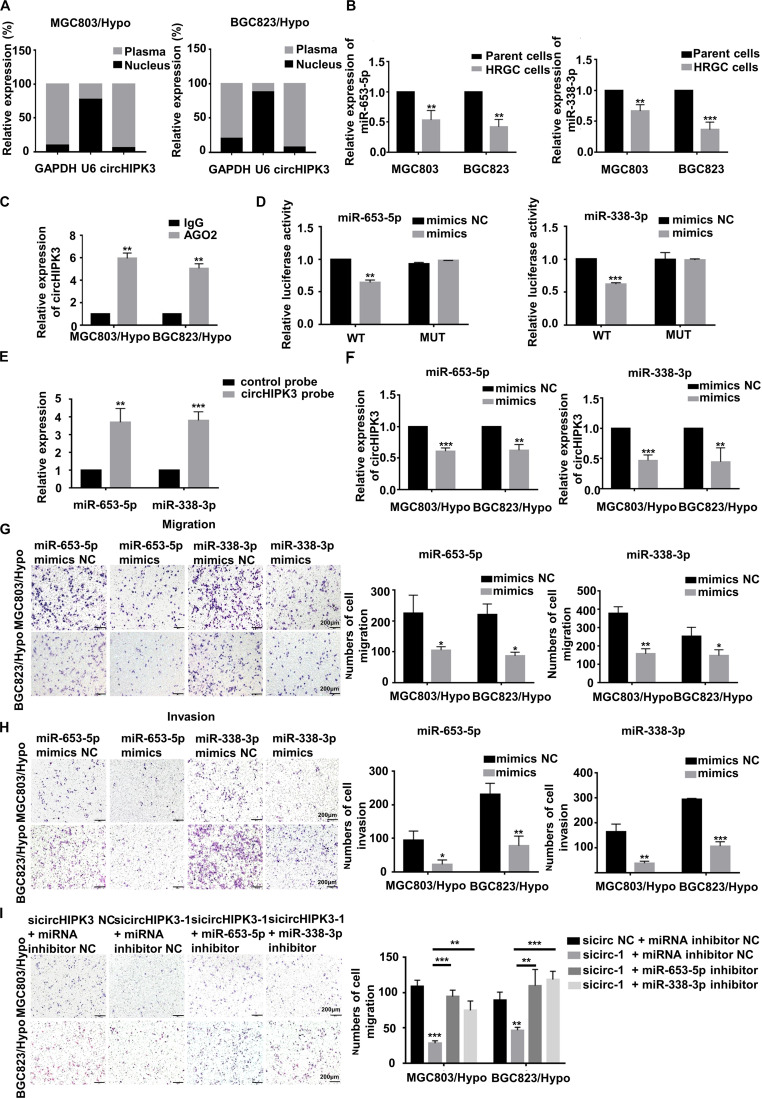
CircHIPK3 promoted migration and invasion of HRGC cells by sponging with miR-653-5p and miR-338-3p. **(A)** The distribution proportion of circHIPK3 in nucleus and cytoplasma of HRGC cells was detected by qRT-PCR. GAPDH and U6 were used as internal controls. **(B)** The relative expression of miR-653-5p and miR-338-3p in HRGC cells compared with their parent GC cells was detected by qRT-PCR. U6 was used as an internal control. **(C)** The relative expression of circHIPK3 combined with AGO2 was examined by Anti-AGO2 RIP assay. IgG was used as a negative control. **(D)** The luciferase activities of HRGC cells after co-transfected with luciferase reporter vectors circHIPK3-WT or circHIPK3-Mut and miR-653-5p or miR-338-3p mimics or miR-NC were examined. **(E)** The relative expression of miR-653-5p or miR-338-3p pulled down by circHIPK3 probe was detected by qRT-PCR. **(F)** The relative expression of circHIPK3 after transfected with miR-NC and miR-653-5p or miR-338-3p mimics. 18S was used as an internal control. **(G,H)** The migration and invasion ability of HRGC cells after transfected with miR-NC and miR-653-5p or miR-338-3p mimics was examined by transwell assay (original magnification, 100×). The columns on the right are quantified by counting 3 fields, and presented as the mean ± standard deviation. *p < 0.05, **p < 0.01, ***p < 0.001. **(I)** The migration ability of HRGC cells after co-transfected with siNC or circHIPK3 siRNAs and miR-NC or miR-653-5p or miR-338-3p inhibitor was examined by transwell assay (original magnification, 100×). The columns on the right are quantified by counting 3 fields, and presented as the mean ± standard deviation. *p < 0.05, **p < 0.01, ***p < 0.001. Data are presented as the mean ± SD of three independent experiments. *p < 0.05, **p < 0.01, ***p < 0.001.

It is known that the cellular localization of circRNAs was closely related to their functions. Therefore, to clarify the molecular mechanism of action of circHIPK3 on long-term hypoxia-promoting metastasis, the expression of circHIPK3 in nucleus and cytoplasm was examined separately by qRT-PCR assay. The result demonstrated that circHIPK3 was principally enriched in the cytoplasm (Figure 3A), indicating its feasibility as a miRNA sponge function. Next, underlying targeted miRNAs of circHIPK3 were predicted using three databases: circBank^1^, Circular RNA Interactome^2^ and StarBase V2.0^3^. As a result, two miRNAs (miR-653-5p and miR-338-3p) with more than four binding sites with circHIPK3, were predicted on all three websites (Supplementary Figure S1A). Then, the sponging relationship between circHIPK3 and miR-653-5p or miR-338-3p was verified in HRGC cells. The result revealed that miR-653-5p and miR-338-3p in HRGC cells were both lower than that in their parent GC cells (Figure 3B). Considering Argonaute2 (AGO2) protein, binding with circRNAs and miRNAs, is the core of RNA-induced silencing complex (RISC), an RIP assay was performed to confirm that anti-AGO2 could enrich more circHIPK3, miR-653-5p, and miR-338-3p molecules compared to anti-IgG under a long-term hypoxic microenvironment (Supplementary Figure S1B and Figure 3C). Furthermore, miR-653-5p and miR-338-3p mimics significantly reduced the luciferase activity of wild-type circHIPK3 but not mutant-type circHIPK3 (Supplementary Figure S1C and Figure 3D). Meanwhile, RNA pull down assay was performed to confirm that miR-653-5p and miR-338-3p could be significantly pulled down by biotinylated probe of circHIPK3 compared to control (Figure 3E). Finally, circHIPK3-KD1 enhanced miR-653-5p and miR-338-3p expression, whereas miR-653-5p and miR-338-3p mimics attenuated circHIPK3 expression, respectively, in HRGC cells (Supplementary Figure S1D and Figure 3F). These results therefore revealed that circHIPK3 could directly combine to miR-653-5p and miR-338-3p in GC under a long-term hypoxic microenvironment.

Next, the function of miR-653-5p and miR-338-3p in the metastatic process of HRGC cells was investigated. As a result, miR-653-5p and miR-338-3p mimics significantly restrained migration and invasion capability in HRGC cells (Figures 3G,H), indicating the metastatic inhibiting function of these miRNAs. The further to prove the involvement of miR-653-5p and miR-338-3p in circHIPK3-induced metastasis, circHIP3-KD1 and miRNA inhibitors were co-transfected into HRGC cells. As shown in Figure 3I, circHIPK3-KD-inhibiting migration was partially reversed by miR-653-5p or miR-338-3p inhibitors in HRGC cells, further illustrating that circHIPK3 could promote GC metastasis by directly interacting with miR-653-5p and miR-338-3p in GC under a long-term hypoxic microenvironment.

### CircHIPK3 Promoted Migration and Invasion of HRGC Cells via the miR-653-5p/miR-338-3p-NRP1 Axis

**FIGURE 4:**
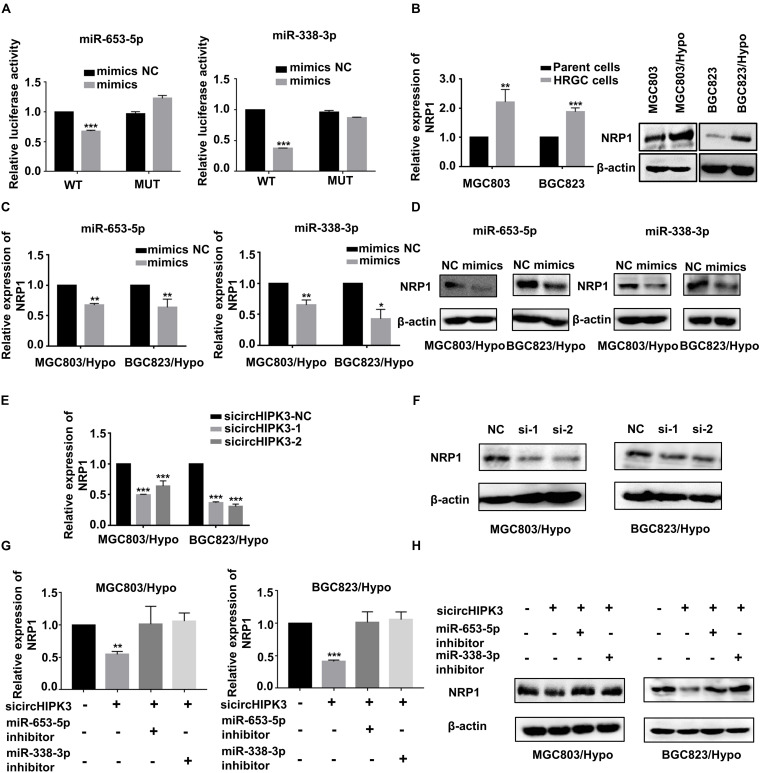
CircHIPK3 promoted migration and invasion of HRGC cells via the miR-653-5p/miR-338-3p-NRP1 axis. **(A)** The luciferase activities of HRGC cells after co-transfected with luciferase reporter vectors NRP1-WT or NRP1-Mut and miR-653-5p or miR-338-3p mimics or miR-NC were examined. **(B)** The relative mRNA and protein expression of NRP1 in HRGC cells compared with their parent GC cells was detected by qRT-PCR and western blot. **(C,D)** The relative mRNA and protein expression of NRP1 in HRGC cells after transfected with miR-NC and miR-653-5p or miR-338-3p mimics was detected by qRT-PCR and western blot. **(E,F)** The relative mRNA and protein expression of NRP1 in HRGC cells after transfected with siNC or circHIPK3 siRNAs was detected by qRT-PCR and western blot. **(G,H)** The relative mRNA and protein expression of NRP1 in HRGC cells after co-transfected with siNC or circHIPK3 siRNAs and miR-NC or miR-653-5p or miR-338-3p inhibitor was detected by western blot. Data are presented as the mean ± SD of three independent experiments. *p < 0.05, **p < 0.01, ***p < 0.001. 18S was used as an internal control for all qRT-PCR experiments. β-actin was used as an internal control for all western blot assays.

To find the target gene of miR-653-5p and miR-338-3p, the miRanda^4^ and TargetScan databases^5^ were applied to predict the common target gene for these two miRNAs. Neuropilin 1 (NRP1), which was known to be involved in metastatic process of cancers, was selected. Dual luciferase reporter assay demonstrated that miR-653-5p and miR-338-3p mimics significantly reduced the luciferase activity of wild-type NRP1 but not mutant-type NRP1, indicating miR-653-5p and miR-338-3p could directly bind to NRP1 (Figure 4A). For further verification, NRP1 expression levels were examined in HRGC cells and parent GC cells, and the result confirmed that NRP1 was upregulated in HRGC cells (Figure 4B). Furthermore, it was shown that the mimics of miR-653-5p and miR-338-3p, as same as circHIPK3-KD, reduced NRP1 expression in MGC803/Hypo and BGC823/Hypo (Figures 4C–F). In addition, circHIPK3-KD1-downregulated NRP1 expression was also partially reversed by co-transfection with miR-653-5p or miR-338-3p inhibitors (Figures 4G,H). Therefore, these data indicated that circHIPK3 upregulated NRP1 expression by sponging with miR-653-5p and miR-338-3p in GC under a long-term hypoxic microenvironment.

### CircHIPK3 Promoted Migration and Invasion of HRGC Cells via the NRP1-ERK/AKT Pathway

**FIGURE 5:**
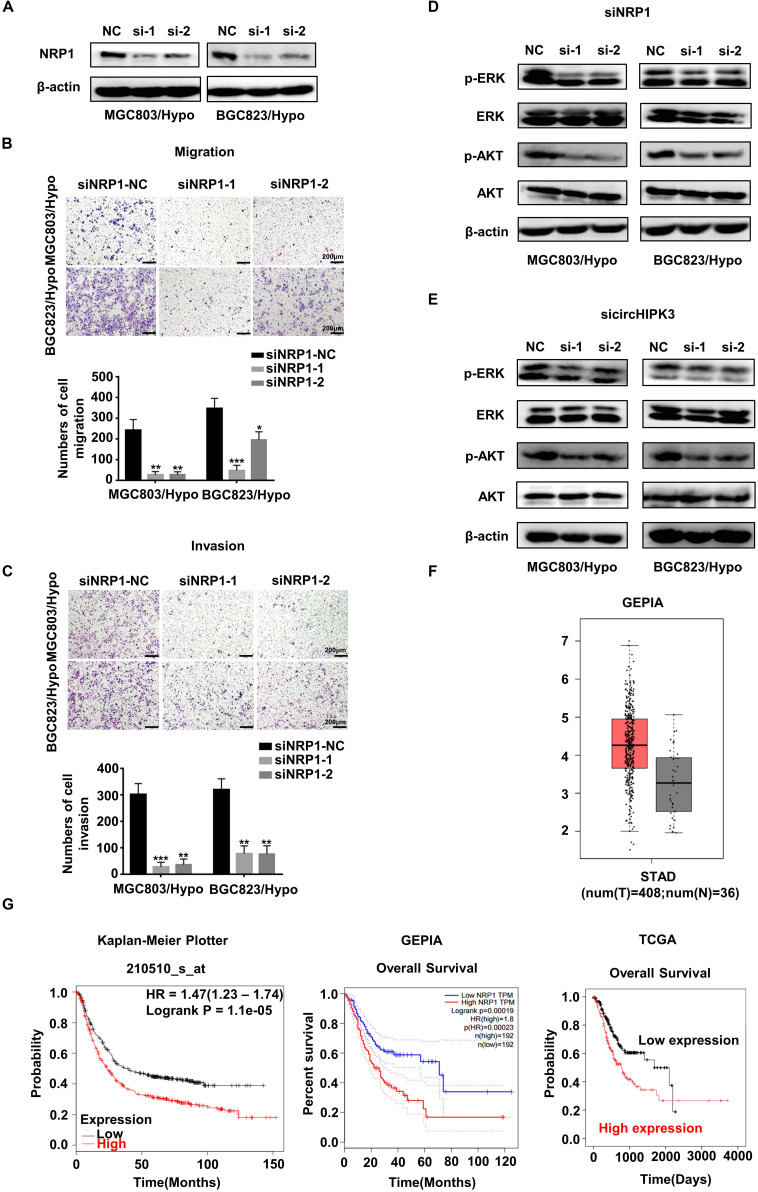
CircHIPK3 promoted migration and invasion of HRGC cells via the NRP1-ERK/AKT pathway. **(A)** The knockdown efficiency of NRP1 in HRGC cells was detected by western blot. **(B,C)** The migration and invasion ability of HRGC cells after transfected with siNC or NRP1 siRNAs was examined by transwell assay (original magnification, 100×). The columns on the down panels are quantified by counting 3 fields, and presented as the mean ± standard deviation. *p < 0.05, **p < 0.01, ***p < 0.001. **(D)** The downstream pathway proteins in HRGC cells after transfected with siNC or NRP1 siRNAs were detected by western blot. **(E)** The same downstream pathway proteins as **(D)** in HRGC cells after transfected with siNC or circHIPK3 siRNAs were detected by western blot. **(F)** The relative expression of NRP1 in GC tissues and adjacent normal tissues was analyzed by GEPIA database. **(G)** The overall survival of GC patients with NRP1-high expression or NRP1-low expression was analyzed by GEPIA, Kaplan-Meier Plotter and TCGA databases. Data are presented as the mean ± SD of three independent experiments. β-actin was used as an internal control for all western blot assays.

The involvement of NRP1 in the metastatic process of HRGC cells was also investigated. The result showed that NRP1-KD not only significantly suppressed the migration and invasion capability of HRGC cells (Figures 5A–C), but also decreased the phosphorylation level of ERK and AKT, in downstream pathways of NRP1 (Figure 5D). A similar result was also obtained using circHIPK3-KD (Figure 5E). The results showed that circHIPK3 could promote migration and invasion via the NRP1-ERK/AKT pathway in HRGC cells. Moreover, the clinical significance of NRP1 was further analyzed using the following on-line databases: GEPIA^6^, Kaplan-Meier Plotter^7^, and TCGA^8^. The result of GEPIA website showed that NRP1 expression significantly increased in GC tissues compared to the adjacent normal tissues (Figure 5F). The Kaplan-Meier Plotter website showed that the overall survival (OS) of GC patients with NPR1-high expression was shorter than that with NPR1-low expression. The GEPIA website and TCGA data analyzed by best cut-off also showed the similar results (Figure 5G), indicating that NRP1 was a poor prognostic biomarker for GC. Taken together, these data demonstrated that circHIPK3-upregulated NRP1 could promote GC metastasis via the ERK/AKT pathway and may lead to poor prognosis of GC patients.

### Verification of the CircHIPK3-miR-653-5p/miR-338-3p-NRP1 Axis in GC Tissues

**FIGURE 6:**
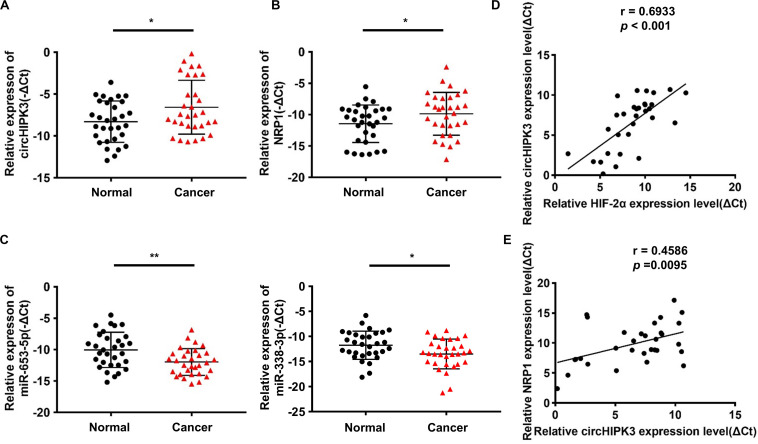
Verification of the circHIPK3-miR-653-5p/miR-338-3p-NRP1 axis in GC tissues. **(A,B)** The relative expression of circHIPK3 and NRP1 in 31 pairs of GC tissues and adjacent non-tumor tissues of patients was detected by qRT-PCR. **(C)** The relative expression of miR-653-3p and miR-338-3p in 31 pairs of GC tissues and adjacent non-tumor tissues of patients was detected by qRT-PCR. **(D)** The correlation between HIF-2α and circHIPK3 expression in GC tissues was analyzed by Pearson correlation analysis. **(E)** The correlation between circHIPK3 and NRP1 expression in GC tissues was analyzed by Pearson correlation analysis. *p < 0.05, **p < 0.01.

The further to confirm the role of the circHIPK3-miR-653-5p/miR-338-3p-NRP1 axis in GC, qRT-PCR was conducted on GC tissues and adjacent normal tissues of 31 GC patients. The results confirmed that circHIPK3 and NRP1 expression was increased, whereas miR-653-5p and miR-338-3p expression was reduced in GC tissues compared with that in adjacent normal tissues (Figures 6A–C); HIF-2α levels were shown to be positively correlated with circHIPK3 levels in GC tissues (Figure 6D); moreover, circHIPK3 mRNA levels were positively correlated with NRP1 mRNA levels (Figure 6E). Therefore, all these data further proved that circHIPK3 was upregulated by HIF-2α and functioned by constructing the ceRNA network with miR-653-5p/miR-338-3-NRP1 under a long-term hypoxic microenvironment in GC.

## Discussion

In this study, we found that circHIPK3, upregulated by HIF-2α, could promote migration and invasion of HRGC cells via the miR-653-5p/miR-338-3p-NPR1 axis, indicating that circHIPK3 participated in metastatic promotion of GC under a long-term hypoxic microenvironment.

Hypoxia, an important typical characteristic of solid malignant tumors, often leads to poor prognosis of cancer by contributing to metastasis. Hypoxia can be divided into acute hypoxia and chronic hypoxia based on the dynamics of oxygen deprivation: the real status of the hypoxic microenvironment inside solid tumors is closer to chronic hypoxia, or so-called long-term hypoxia, rather than acute hypoxia (25). The HRGC cell lines in this study established in our laboratory previously have been shown to be a good model for long-term hypoxia-related research in GC. Using these HRGC cells, we have revealed that LncRNA—UCA1 was upregulated, and promoted the migration of HRGC cells through the miR-7-5p/EGFR axis under long-term hypoxia (14). Now, we have further demonstrated that circRNA—circHIPK3 was also increased in HRGC cells and promoted GC metastasis under a long-term hypoxic microenvironment. CircHIPK3, a classical circular RNA involved in cancer development, appeared to play opposite roles in different cancers. CircHIPK3 promoted proliferation, metastasis, and chemotherapy resistance in lung cancer, colorectal cancer, and prostate cancer, whereas it suppressed cell proliferation, migration, and invasion in osteosarcoma (20, 21, 26, 27). However, only three studies on circHIPK3 were reported in GC, and the conclusions were still contradictory. The contradiction might be due to the strong heterogeneity of GC resulting in the inconsistent effect of circHIPK3 in different specimens. In our study, we found that overexpression of circHIPK3 in normoxia could promote metastasis of GC and the expression of circHIPK3 increased in GC tissues compared with that in adjacent normal tissues, indicating circHIPK3 might play an oncogenic role in GC. Our findings that circHIPK3 was upregulated in HRGC cells and promoted GC metastasis, might reflect the heterogeneity of GC because of hypoxia, and partially explain the different roles of circHIPK3 in GC as evinced by our result and previous studies. Certainly, many other factors, such as the number of samples, sampling quality, tumor cell content, storage conditions and time, RNA extraction, qRT-PCR and etc., may also lead to this contradictory conclusion. In the future, more GC samples are needed to collect further to investigate the definite roles of circHIPK3 in GC.

Hypoxia-inducible factors are the key transcriptional regulatory factors of many target genes in hypoxia (28). It is known that HIF-1α exhibits stable expression and plays the main transcriptional role in acute hypoxia, while HIF-2α is also stable but mainly functions in chronic hypoxia (25). Although HIF-1α and HIF-2α could both promote target gene transcription by combining with the HRE promoter region, their target genes are not completely consistent (29–31). For example, HE4 and RIT1 can only be transcriptionally regulated by HIF-1α, while LncNEAT1 and PTPMT1 can only be transcriptionally regulated by HIF-2α (32–35). In this study, HIF-2α-KD, but not HIF-1α-KD, decreased circHIPK3 expression, and the strong positive correlation was verified between HIF-2α and circHIPK3 in GC samples, indicating that circHIPK3 is a novel target of HIF-2α. Certainly, it still remains unclear whether circHIPK3 is directly upregulated by HIF-2α transcription or is upregulated by another HIF-2α target gene. Further study is warranted in the future.

The localization of circRNAs is essential to their function, and a non-negligible function of circRNAs distributed in cytoplasma is working as sponges by binding with miRNAs (36, 37). CircRNAs can not only sponge with multiple miRNAs but also sponge with the same miRNA at several binding sites. The more miRNAs bound by one kind of circRNAs, the stronger functions of circRNAs in cells. The most typical representative circRNA is ciRS-7, which exists at over 70 binding sites of miR-7 and promotes cancer progression in esophageal squamous cell carcinoma and non-small cell lung cancer (38, 39). In our research, we found that circHIPK3 was principally enriched in cytoplasm of HRGC cells and could combine to miR-653-5p and miR-338-3p with four binding sites, respectively, suggesting the importance of the role of circHIPK3. Besides, qRT-PCR results revealed that the levels of miR-653-5p and miR-338-3p were decreased in HRGC cells, and both of these miRNAs could restrain the migration and invasion of HRGC cells, which was similar to previous research findings indicating that miR-653-5p could suppress growth and invasion in non-small cell lung cancer, and miR-338-3p could suppress tumor progression in colorectal cancer and breast cancer (40–42). Therefore, our research proved that circHIPK3 had an essential effect in facilitating GC metastasis by sponging with miR-653-5p and miR-338-3p under a long-term hypoxic microenvironment.

Neuropilin 1 is a kind of non-tyrosine kinase transmembrane glycoprotein known as a co-receptor of VEGF (43). It was reported that NRP1 could play important role in tumor progression by promoting angiogenesis, proliferation, metastasis, and drug resistance in several different types of cancers (44–47). In this study, based on the result predicted by bioinformatics analysis that NRP1 has stable binding sites with miR-653-5p and miR-338-3p, NRP1 was selected as the common downstream target gene, and the result proved miR-653-5p and miR-338-3p mimics downregulated NRP1 expression, further confirmed this prediction. Although the study of NRP1 in GC remained limited, it was reported that the high expression of NRP1 due to hypomethylation was co-expressed with PDGFRB and was significantly correlated with tumor malignant phenotypes with poor prognosis (48). Similarly, we also found that NRP1-KD restrained the migration and invasion capability of HRGC cells, and NRP1 was involved in circHIPK3 promotion of HRGC metastasis by the sponging with miR-653-5p and miR-338-3p, suggesting the metastatic promotion role of NRP1 in GC under a long-term hypoxic microenvironment. As it is known that NRP1 could activate the MAPK and AKT pathways by binding to VEGF, we also detected the possible downstream pathway of NRP1 in HRGC cells, and found that either NRP1-KD or circHIPK3-KD reduced the expression of *p*-ERK and *p*-AKT, suggesting that NRP1 upregulated by circHIPK3 promoted GC metastasis by activating ERK and AKT pathways in a long-term hypoxic microenvironment (49).

The circRNA-miRNA-mRNA ceRNA network analyzed in this research is composed of circHIPK3, miR-653-5p, and miR-338-3p, each of which have four binding sites with circHIPK3, and NRP1, which is the common target gene of the two miRNAs. Therefore, long-term hypoxia-upregulated circHIPK3 significantly promoted GC metastasis via construction of a stable ceRNA network with miR-653-5p/miR-338-3-NRP1, indicating the important functions of circHIPK3 in GC metastasis under a long-term hypoxic microenvironment. In our study, the stable ceRNA network was also verified in GC tissues and obtained similar results with that in GC cells. However, due to limited GC samples, it needs to be verified in larger scale samples in the future. Certainly, other mechanisms of circHIPK3 except for the ceRNA function under a long-term hypoxic microenvironment of GC also needs the further exploration.

**FIGURE 7:**
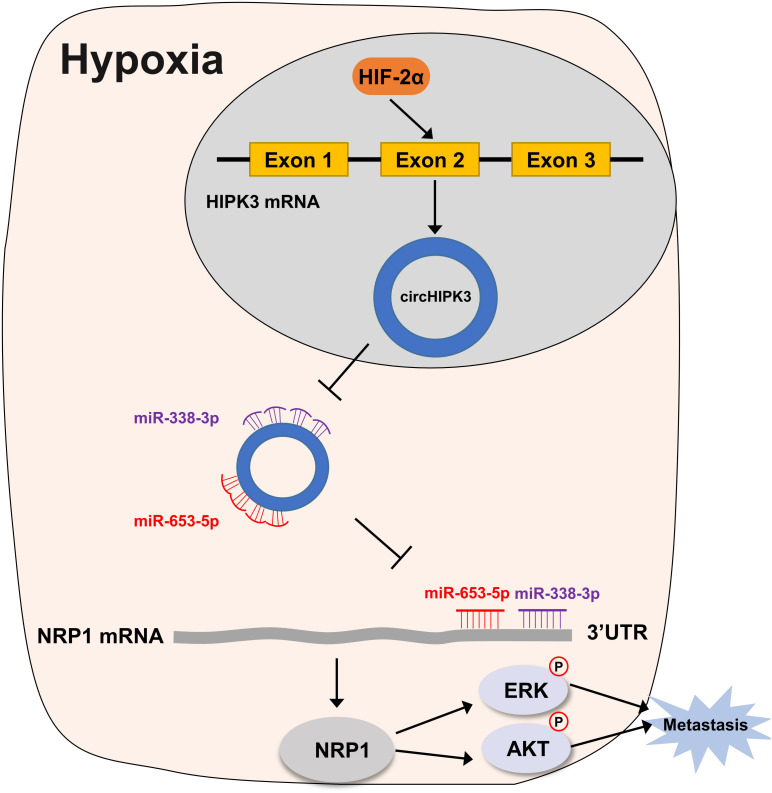
Working model for circHIPK3 in HRGC cells. Under a long-term hypoxic microenvironment of gastric cancer, circHIPK3 was upregulated by HIF-2α; then circHIPK3 upregulated NRP1 by sponging with miR-653-3p and miR-338-3p followed by relieving the transcriptional suppress of NRP1; finally, NRP1 promoted metastasis by activating ERK and AKT pathway.

In summary, our study demonstrated that circHIPK3 upregulated by HIF-2α could facilitate the migration and invasion of GC cells via the miR-653-5p/miR-338-3p-NRP1 axis under a long-term hypoxic microenvironment (the mechanism is shown in diagrammatic form in Figure 7. These findings revealed a new mechanism of long-term hypoxia-promoting metastasis in GC and showed that circHIPK3 might be a long-term hypoxic biomarker and a potential prognostic biomarker for GC patients in the future.

## Data Availability Statement

All datasets presented in this study are included in the article/Supplementary Material.

## Ethics Statement

The studies involving human participants were reviewed and approved by the Ethics Committee of the First Hospital of China Medical University. The patients/participants provided their written informed consent to participate in this study.

## Author Contributions

YL and XC designed the research study. YJ did the majority of the experiment and wrote the manuscript. WL and YW analyzed the data. XQ, KH, JW, CL, and XZ conducted the experimental guidance. JZ and XL contributed essential samples. YL, JZ, and XC revised the manuscript. All authors reviewed and approved the final manuscript.

## Conflict of Interest

The authors declare that the research was conducted in the absence of any commercial or financial relationships that could be construed as a potential conflict of interest.

## Footnotes

1
http://www.circbank.cn/

2
https://circinteractome.nia.nih.gov/

3
http://starbase.sysu.edu.cn/

4
http://www.microrna.org/microrna/home.do

5
http://www.targetscan.org/vert_72/

6
http://gepia.cancer-pku.cn/detail.php

7
http://kmplot.com/analysis/

8
https://portal.gdc.cancer.gov

## Abbreviations

GC: gastric cancer
HIF: hypoxia-inducible factor
HRGC: hypoxic resistant gastric cancer
KD: knockdown
NC: negative control
NRP1: neuropilin 1
PVDF: polyvinylidene difluoride
qRT-PCR: quantitative real-time PCR
TCGA: the Cancer Genome Atlas.
