# Corrigendum: CircHIPK3 Promotes Metastasis of Gastric Cancer *via* miR-653-5p/miR-338-3p-NRP1 Axis Under a Long-Term Hypoxic Microenvironment

**DOI:** 10.3389/fonc.2021.783320

**Published:** 2021-11-12

**Authors:** Yue Jin, Xiaofang Che, Xiujuan Qu, Xin Li, Wenqing Lu, Jie Wu, Yizhe Wang, Kezuo Hou, Ce Li, Xiaojie Zhang, Jianping Zhou, Yunpeng Liu

**Affiliations:** ^1^ Department of Medical Oncology, The First Hospital of China Medical University, Shenyang, China; ^2^ Key Laboratory of Anticancer Drugs and Biotherapy of Liaoning Province, The First Hospital of China Medical University, Shenyang, China; ^3^ Liaoning Province Clinical Research Center for Cancer, Shenyang, China; ^4^ Key Laboratory of Precision Diagnosis and Treatment of Gastrointestinal Tumors, Ministry of Education, Shenyang, China; ^5^ Department of Gastrointestinal Surgery, The First Hospital of China Medical University, Shenyang, China

**Keywords:** CircHIPK3, long-term hypoxic microenvironment, HIF-2α, gastric cancer, metastasis

In the original article, there was a mistake in **Figure 2D** as published. The picture of migration of sicircHIPK3 in BGC823/Hypo cells in **Figure 2D** was misused. The corrected **Figure 2** appears below.

The authors apologize for this error and state that this does not change the scientific conclusions of the article in any way. The original article has been updated.

**Figure 2:**
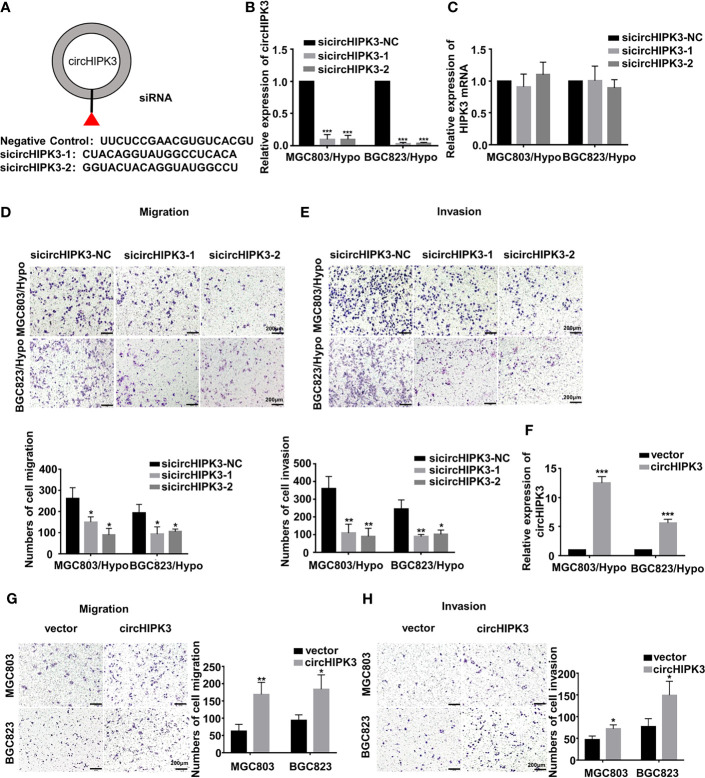
CircHIPK3 promoted migration and invasion of HRGC cells. **(A)** The sequence of two siRNAs targeted to back-splicing site of circHIPK3 and the negative control siRNA. **(B, C)** The relative expression of circHIPK3 and linear HIPK3 mRNA in HRGC cells after transfected with negative control siRNA (siNC) or circHIPK3 siRNAs was detected by qRT-PCR. 18S was used as an internal control. **(D, E)** The migration and invasion ability of HRGC cells after transfected with siNC or circHIPK3 siRNAs was examined by transwell assay (original magnification, 100×). The columns on the down panels are quantified by counting 3 fields, and presented as the mean ± standard deviation. *p < 0.05, **p < 0.01, ***p < 0.001. **(F)** The overexpression efficiency of circHIPK3 in MGC803 and BGC823 cells was detected by qRT-PCR. 18S was used as an internal control. **(G, H)** The migration and invasion ability of MGC803 and BGC823 cells after transfected with circHIPK3 overexpression plasmids and empty vectors was examined by transwell assay (original magnification, 100×). The columns on the right are quantified by counting three fields, and presented as the mean ± standard deviation. *p < 0.05, **p < 0.01, ***p < 0.001. Data are presented as the mean ± SD of three independent experiments. *p < 0.05, **p < 0.01, ***p < 0.001.

## Publisher’s Note

All claims expressed in this article are solely those of the authors and do not necessarily represent those of their affiliated organizations, or those of the publisher, the editors and the reviewers. Any product that may be evaluated in this article, or claim that may be made by its manufacturer, is not guaranteed or endorsed by the publisher.

