# Supplementary material for: CircHIPK3 Promotes Metastasis of Gastric Cancer via miR-653-5p/miR-338-3p-NRP1 Axis Under a Long-Term Hypoxic Microenvironment
Source: Front Oncol. 2020 Aug 13;10:1612. doi: 10.3389/fonc.2020.01612 (PMC7443574; doi:10.3389/fonc.2020.01612)
Supplement: Supplementary file 1 [file Data_Sheet_1.PDF]

Supplementary Material

Supplementary Table 1

The sequences of primers and siRNA used in this study

**The sequences of primers for PCR**	
HIPK3 mRNA	Forward: TGGAGACTGGGGGAAGATGA
	Reverse: CACACTAACTGGCTGAGGGG
CircHIPK3	Forward: TATGTTGGTGGATCCTGTTCGGCA
	Reverse: TGGTGGGTAGACCAAGACTTGTGA
NRP1	Forward: GAAAAATGCGAATGGCTGAT
	Reverse: AATGGCCCTGAAGACACAAC
HIF-1α	Forward: CTCAAAGTCGGACAGCCTCA
	Reverse: CCCTGCAGTAGGTTTCTGCT
HIF-2α	Forward: GTGACATGATCTTTCTGTCGGAA
	Reverse: CGCAAGGATGAGTGAAGTCAAA
18s RNA	Forward: CCCGGGGAGGTAGTGACGAAAAAT
	Reverse: CGCCCGCCCGCTCCCAAGAT
U6	Forward: GCTTCGGCAGCACATATACTAAAAT
	Reverse: CGCTTCACGAATTTGCGTGTCAT
GAPDH	Forward: ACCCACTCCTCCACCTTTGAC
	Reverse: TGTTGCTGTAGCCAAATTCGTT
miR-653-5p	GGTGCTGAAACAATCTCTACTGAAA
miR-338-3p	CACCATCCTGCTCCAGTGTT

**The sequences of siRNAs**	
NC	UUCUCCGAACGUGUCACGU
sicircHIPK3-1	CUACAGGUAUGGCCUCACA
sicircHIPK3-2	GGUACUACAGGUAUGGCCU
siNRP1-1	GGUAUGGUGUCUGGACUU
siNRP1-2	GCGAUACUAUAAAAAUUG
miR-653-5p mimics	GUGUUGAAACAAUCUCUACUG
miR-653-5p inhibitor	GUGUUGAAACAAUCUCUACUG
miR-338-3p mimics	UCCAGCAUCAGUGAUUUUGUUG
miR-338-3p inhibitor	UCCAGCAUCAGUGAUUUUGUUG

Supplementary Figure 1

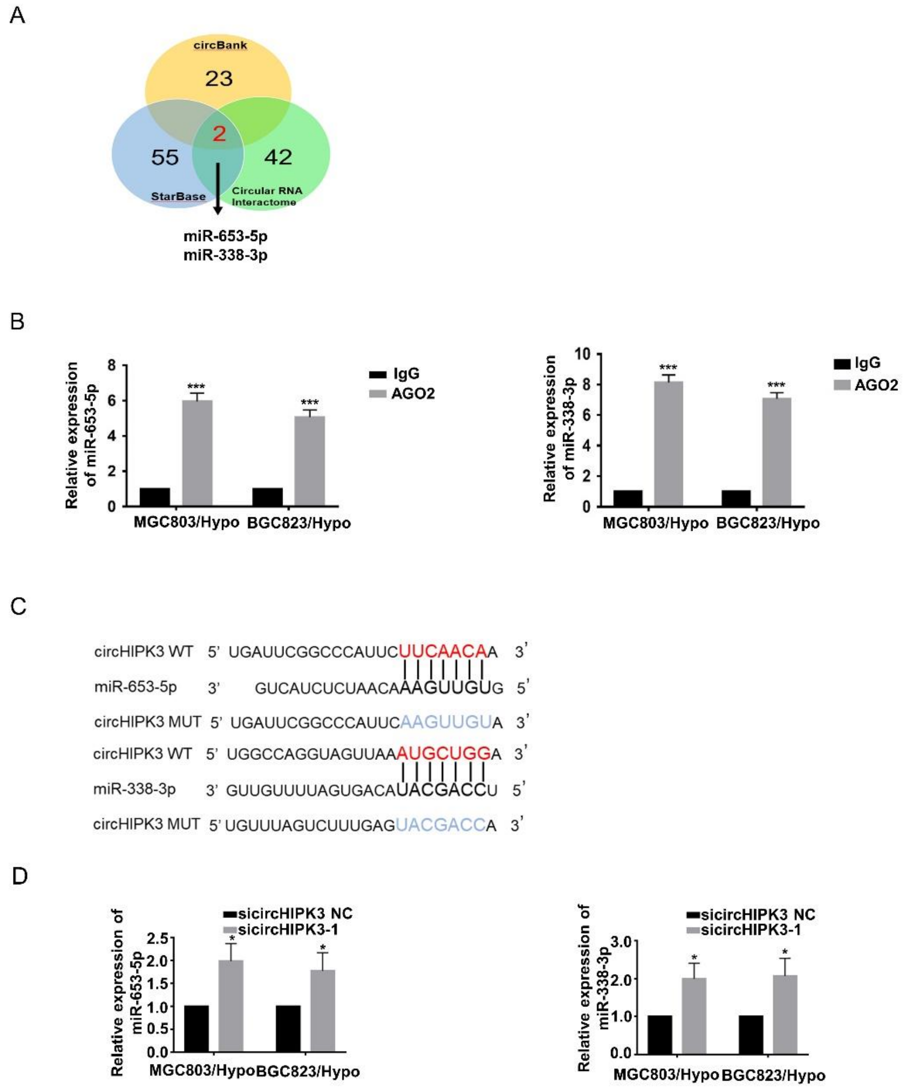

Supplementary Figure 1. (A) The target miRNAs predicted by circBank, StarBase and Circular RNA Interactome databases were shown in the Venn Diagram. (B) The relative expression of miR-653-5p and miR-338-3p combined with AGO2 was examined by Anti-AGO2 RIP assay. IgG was used as a negative control. (C) The binding sites between circHIPK3 and miR-653-3p or miR-338-3p were shown. (D) The relative expression of miR-653-5p and miR-338-3p in HRGC cells after transfected with siRNA NC or circHIPK3 siRNAs was detected by qRT-PCR. U6 was used as an internal control.
